# Modulation of Iron Import and Metronidazole Resistance in *Bacteroides fragilis* Harboring a *nimA* Gene

**DOI:** 10.3389/fmicb.2022.898453

**Published:** 2022-06-09

**Authors:** Ana Paunkov, József Sóki, David Leitsch

**Affiliations:** ^1^Institute for Specific Prophylaxis and Tropical Medicine Center for Pathophysiology, Infectiology, and Immunology, Medical University of Vienna, Vienna, Austria; ^2^Faculty of Medicine, Institute of Medical Microbiology, University of Szeged, Szeged, Hungary

**Keywords:** *Bacteroides fragilis*, metronidazole, *nim* genes, resistance, iron import

## Abstract

*Bacteroides fragilis* is a commensal of the human gut but can also cause severe infections when reaching other body sites, especially after surgery or intestinal trauma. *Bacteroides fragilis* is an anaerobe innately susceptible to metronidazole, a 5-nitroimidazole drug that is prescribed against the majority of infections caused by anaerobic bacteria. In most of the cases, metronidazole treatment is effective but a fraction of *B*. *fragilis* is resistant to even very high doses of metronidazole. Metronidazole resistance is still poorly understood, but the so-called *nim* genes have been described as resistance determinants. They have been suggested to encode nitroreductases which reduce the nitro group of metronidazole to a non-toxic aminoimidazole. More recent research, however, showed that expression levels of *nim* genes are widely independent of the degree of resistance observed. In the search for an alternative model for *nim*-mediated metronidazole resistance, we screened a strain carrying an episomal *nimA* gene and its parental strain 638R without a *nim* gene for physiological differences. Indeed, the 638R daughter strain with the *nimA* gene had a far higher pyruvate-ferredoxin oxidoreductase (PFOR) activity than the parental strain. High PFOR activity was also observed in metronidazole-resistant clinical isolates, either with or without a *nim* gene. Moreover, the strain carrying a *nimA* gene fully retained PFOR activity and other enzyme activities such as thioredoxin reductase (TrxR) after resistance had been induced. In the parental strain 638R, these were lost or very strongly downregulated during the development of resistance. Further, after induction of high-level metronidazole resistance, parental strain 638R was highly susceptible to oxygen whereas the daughter strain with a *nimA* gene was hardly affected. Ensuing RT-qPCR measurements showed that a pathway for iron import *via* hemin uptake is downregulated in 638R with induced resistance but not in the resistant *nimA* daughter strain. We propose that *nimA* primes *B*. *fragilis* toward an alternative pathway of metronidazole resistance by enabling the preservation of normal iron levels in the cell.

## Introduction

*Bacteroides fragilis* is a human anaerobic gut commensal and a member of the large genus *Bacteroides* which accounts for ≈30% of the human fecal isolates (Kuwahara et al., 2004). *Bacteroides* spp. ferment carbohydrates and produce volatile short-chain fatty acids which are absorbed by the large intestine (Wexler, 2007). Despite their primarily beneficial role, members of the genus *Bacteroides* can also occasionally cause severe disease, mainly in the course of injuries of the gastrointestinal tract (Aldridge and Sanders, 2002). They are mainly involved in mixed aerobic–anaerobic infections leading to abscesses in organs, but they can also cause bacteremia (Wexler, 2007). Although *B*. *fragilis* represents less than one percent of the intestinal microbiome, it alone accounts for as much 30–60% of all clinical isolates from the large intestine (Wexler, 2007; Tan et al., 2017). Indeed, *B*. *fragilis* expresses a metalloprotease as a toxin, termed fragilysin, and is capable of digesting E-cadherin at the intestinal epithelium's tight junctions (Remacle et al., 2014; Yekani et al., 2020) which might explain its eminent role as a pathogen within the genus *Bacteroides*.

Currently, the most reliable treatment options for *B*. *fragilis* infections are carbapenems and metronidazole due to low resistance rates (Snydman et al., 2017). Metronidazole is a 5-nitroimidazole drug which was specifically developed for the treatment of infections with the anaerobic parasite *Trichomonas vaginalis* (reviewed in Leitsch, 2019) but has also been found effective against most anaerobic pathogens, including *B. fragilis*. Metronidazole as such is a prodrug which needs to be reduced at its nitro group in order to exert toxicity. It has still not been completely resolved in which intermediate is responsible for the toxic effect, but the respective nitroimidazole anion and the respective nitroso radical, generated through a single or double electron transfer to the nitro group, respectively, are the most probable candidates. DNA has been proposed as the major target of metronidazole, but it also forms adducts with cysteines leading to oxidative damage (reviewed in Leitsch, 2019).

Although resistance to metronidazole in *B*. *fragilis* is, in general, still comparably rare (Snydman et al., 2017), it occurs much more often in some parts of the world and can reach up to 10% of all cases treated (Vieira et al., 2006; Yehya et al., 2014; Sheikh et al., 2015). Several resistance mechanisms have been proposed: i., loss of enzyme pathways leading to metronidazole reduction (Narikawa, 1986), ii., efflux pumps (Pumbwe et al., 2006, 2007), and iii., Nim protein-mediated resistance (Alauzet et al., 2019). The most obvious candidate for a metronidazole-reducing enzyme is pyruvate:ferredoxin oxidoreductase (PFOR) which exists basically in all microorganisms susceptible to metronidazole (Narikawa, 1986) and which can reduce metronidazole *in vitro via* ferredoxin (reviewed in Leitsch, 2019). Indeed, PFOR is downregulated in most organisms after induction of metronidazole resistance *in vitro* (reviewed in Leitsch, 2019) but the deletion of PFOR has only minimal effect on metronidazole susceptibility in *B*. *fragilis* (Diniz et al., 2004). The overexpression of efflux pumps of the RND class reduces susceptibility to metronidazole by about 2-fold (Pumbwe et al., 2006, 2007), but this is still well below the breakpoint value for metronidazole resistance of 4 μg ml^−1^ according to EUCAST (The European Committee on Antimicrobial Susceptibility Testing. Breakpoint tables for interpretation of MICs and zone diameters. Version 12.0, 2022. http://www.eucast.org). Finally, *nim* genes were identified as transmissible, mainly plasmid-borne, determinants of metronidazole resistance (Breuil et al., 1989; Sebald, 1994) and were proposed to encode nitroreductases which can reduce the nitro group of metronidazole to a non-reactive amino group through the transfer of six electrons (Carlier et al., 1997). Currently, 11 types of *nim* genes are known (*nimA* to *nimK*) but all are assumed to have an identical function (Alauzet et al., 2019). Surprisingly, only a proportion of strains carrying *nim* genes are indeed resistant (Löfmark et al., 2005), that is, the level of resistance conferred by *nim* genes is normally below the breakpoint concentration for metronidazole. High-level resistance, however, can be induced much more quickly in strains with a *nim* gene (Gal and Brazier, 2004; Löfmark et al., 2005; Leitsch et al., 2014). In contrast to the notion of Nim proteins acting as nitroreductases, however, expression levels of Nim were not found elevated in the resistant strains but remained unchanged (Leitsch et al., 2014). This rather argues against Nim proteins acting directly as nitroreductases and suggests a more complementary and indirect effect, possibly by affecting the cellular physiology.

In this study, we aimed at identifying such indirect effect of *nim* genes on metronidazole susceptibility by performing comparative physiological screenings of strains carrying an episomal *nim* gene and their respective parental strain without a *nim* gene. In addition, two clinical metronidazole-resistant strains, one with, another without a *nim* gene, were studied for comparison. We assayed the activities of major metabolic enzymes, that is, PFOR, lactate dehydrogenase (LDH), malate dehydrogenase (MDH), and fumarate reductase (FR), and of central antioxidant enzymes, that is, catalase, superoxide dismutase (SOD), and thioredoxin reductase (TrxR), before and after induction of high-level metronidazole resistance. Further, sensitivity to oxygen was measured in all strains.

## Materials and Methods

### Chemicals and Growth Media Components

Wilkins-Chalgren anaerobe agar (WC) was purchased from Oxoid (Basingstoke, England), and Brain Heart Infusion Broth (BHI), Brain Heart Infusion Agar, and vitamin K1 were purchased from Carl Roth (Karlsruhe, Germany). Hemin, metronidazole, NADH, NADPH, cytochrome c, benzyl viologen dichloride, catalase from bovine liver, paraquat dichloride hydrate, Tris/HCl, Triton X-100, xanthine, pyruvic acid, oxaloacetate, sodium fumarate, β-mercaptoethanol, and Coenzyme A were all purchased from Sigma-Aldrich (St. Luis, USA). Potassium dihydrogen phosphate (KH_2_PO_4_), hydrogen peroxide, sodium dithionite, xanthine oxidase, ethylenediaminetetraacetic acid (EDTA), sodium chloride, and Anaerocult A were purchased from Merck (Darmstadt, Germany). Etests were purchased from bioMérieux (Marcy-l'Étoile, France).

### Bacterial Strains and Culture

All strains used in this study originate from the bacterial strain repository of the Institute of Medical Microbiology at the University of Szeged, Hungary. Strain *B*. *fragilis* 638R and two transconjugant daughter strains, one with a *nimA* gene located on plasmid pI417 (Breuil et al., 1989), and another with a *nimE* gene located on plasmid pBF388c (Sóki et al., 2006), were used in this study. The *nimA* gene is positioned behind insertion element IS1168 (Haggoud et al., 1994), whereas the *nimE* gene is positioned behind insertion element IS*Bf* 6 (Sóki et al., 2006). These transconjugants are being referred to as 638R *nimA* and 638R *nimE*, respectively. The sequences of IS1168 and IS*Bf* 6 and the downstream *nim* genes can be accessed *via* the GenBank numbers X71444 and AM042593, respectively. Strain *B*. *fragilis* R19811 is a multidrug-resistant isolate from the UK with high-level metronidazole resistance (Wareham et al., 2005; Paunkov et al., 2022) but without a known *nim* gene (Terhes et al., 2001). Strain *B*. *fragilis* 388/1 is a multidrug-resistant isolate from Kuwait displaying high-level metronidazole resistance (Jamal et al., 2004; Paunkov et al., 2022). It harbors plasmid pBF388c which was used to generate the 638R *nimE* transconjugant (Sóki et al., 2004). The *B*. *fragilis* strains E65, 2294 (Nagy et al., 2001), 30370, and NCTC9343 (ATCC 25285) were used for PFOR activity measurements only: the strains 638R, E65, NCTC9343, and 30370 group into division I (*cfiA*-negative), whereas R19811, 388/1, and 2294 group into division II (*cfiA*-positive) (Nagy et al., 2011). The MICs of all strains for metronidazole as determined by Etest were the following: 638R, 0.25 μg ml^−1^ (Paunkov et al., 2022); 638R *nimA*, 1.5 μg μg ml^−1^ (Paunkov et al., 2022); R19811, 48 μg ml^−1^ (Paunkov et al., 2022); 388/1, 256 μg ml^−1^ (Paunkov et al., 2022); E65, 0.25 μg ml^−1^ (this study), 2294, 0.50 μg ml^−1^ (this study); 30370, 0.38 μg ml^−1^ (this study); NCTC9343, 0.50 μg ml^−1^ (this study).

Cells were grown on either WC agar plates or BHI agar plates with hemin and vitamin K1 supplementation. When cells were needed for assays, they were grown in 14-mL sterile, round bottom, two-position vent stopper tubes (Greiner Bio-One) in supplemented BHI medium (1 μg ml^−1^ vitamin K1 and 5 μg ml^−1^ hemin under anaerobic conditions provided inside anaerobic jars (Merck, Darmstadt, Germany) using the Anaerocult A system (0% O_2_ and 18% CO_2_) at 37°C.

### Induction of Metronidazole Resistance in *B*. *fragilis* 638R Strain With or Without *nimA* Gene

Metronidazole resistance was induced by passaging 638R and 638R *nimA* on WC agar plates with the metronidazole concentration in the plates increasing by factor 2 with every passage. Metronidazole concentrations used were 0.5, 1, 2, 4, 8, 16, 32, and 64 μg ml^−1^. Plates were incubated until copious growth was visible.

The original MICs to metronidazole were 0.25 μg ml^−1^ in 638R (Paunkov et al., 2022) and 1.5 μg ml^−1^ in 638R *nimA* (Paunkov et al., 2022).

### PFOR Activity Assay

PFOR activity was assayed along the lines of a preexisting protocol (Lindmark and Müller, 1973) in 1 ml reaction buffer containing 10 mM paraquat dichloride hydrate, 100 mM KH_2_PO_4_ pH 6.75, 250 mM β-mercaptoethanol, 2.5 mM sodium pyruvate, 0.1% Triton X-100, and 0.25 mM coenzyme A which had been incubated for 1 h under anaerobic conditions provided inside an anaerobic workstation (BugBox, Baker Ruskinn Technology Ltd) at 37°C prior to the measurements. For every measurement, 10^5^ cells, suspended in 50 μl 100 mM tris pH 7.5, were added to the reaction buffer inside the cuvette and sealed with two layers of Parafilm™ in order to prevent influx of oxygen during the transfer into the UV/Vis spectrophotometer (Perkin-Elmer Lambda 25). PFOR activity was measured at λ = 600 nm over the period of 2 min at room temperature.

### Lactate Dehydrogenase (LDH) and Malate Dehydrogenase (MDH) Activity Assay

The activities of LDH and MDH were measured spectrophotometrically by monitoring oxidation of NADH linked to the reduction in pyruvate (LDH) and oxaloacetic acid (MDH), respectively, at λ = 340 nm over the period of 2 min at room temperature. The reaction buffers contained 100 mM Tris/HCl pH 7.5, 0.2 mM NADH, 0.1% Triton X-100, and either 2 mM sodium pyruvate or 2 mM oxaloacetic acid. 10^5^ cells suspended in 50 μl of 100 mM Tris/HCl pH 7.5 were used for each measurement. Background NADH oxidase activities, as determined by measuring NADH oxidation under the same conditions in the absence of pyruvate and oxaloacetate, were subtracted in order to receive LDH and MDH activities.

### Fumarate Reductase Activity

5 × 10^5^ cells from an overnight culture grown in an anaerobic workstation were harvested by centrifugation at 3000 × *g* for 10 min and resuspended in 250 μl of 40 mM Tris/HCl pH 7. 10^5^ cells were used per reaction. The reaction buffer contained 40 mM Tris/HCl pH 7, 2 mM sodium fumarate, 20 mM NaCl, 2.5 mM benzyl viologen dichloride, 0.2 mM sodium dithionite, and 0.1% Triton X-100. Reaction buffer and cells were both prepared strictly under anaerobiosis. After addition of the cells to the buffer in 1 mL cuvettes, the time point of the discoloration of the reaction buffer was recorded. The time required for discoloration of the buffer was interpreted as being inversely proportional to fumarate reductase activity. Samples that failed to discolor within 90 min were considered negative for fumarate reductase activity.

### Aerobic Survival Assay

The ability of the cells to survive extended oxygen exposure was tested using an aerobic survival assay by growing cells in BHI medium until OD_600_ 0.5 was reached. Serial dilutions ranging from 10^−1^ to 10^−5^ were prepared, and 5 μl of each dilution was spotted onto BHI agar plates with or without supplementation. Cells were exposed to air for 0, 48, 72, and 96 h inside the aerobic incubator at 37°C before anaerobic incubation of 48 h inside anaerobic jars at 37°C.

### Catalase Activity

Catalase activity was measured according to a recently published protocol (Paunkov et al., 2022).

### Superoxide Dismutase Assay

SOD activity was measured spectrophotometrically at λ = 550 nm for 2 min at RT according to an established protocol (McCord and Fridovich, 1969). The reaction buffer contained 0.05 M sodium phosphate buffer pH 7.8, 10 μM cytochrome c, 50 μM xanthine, 0.04 mg ml^−1^ xanthine oxidase, 0.1 mM EDTA, and 10, 50, 100, or 200 μg ml^−1^ of cell extract. Cell extracts were prepared by grinding the frozen pellets of overnight cultures in a porcelain mortar with a pestle. Cell lysates in 0.05 M sodium phosphate buffer pH 7.8 were centrifuged at 4°C for 10 min at 12,000 × *g*. The resulting supernatant was collected and used in the assay. One unit of SOD activity was defined as the amount of cell extract necessary to inhibit reduction in cytochrome c in the assay by 50%. IC_50_ values were calculated using GraphPad Prism 9 software.

### Thioredoxin Reductase (TrxR) Activity in *B*. *fragilis* Cell Extracts

The measurement of TrxR activity in *B*. *fragilis* cell extracts with recombinant *B*. *fragilis* thioredoxin A (TrxA) was performed as described recently (Paunkov et al., 2021).

### Antimicrobial Susceptibility Testing

Antimicrobial susceptibility testing was performed using Etests. Several colonies were picked with a sterile swab, resuspended in 1 × PBS, and streaked onto supplemented BHI agar plates. An inoculum of 1 McFarland equivalent was used according to the instructions of the provider (bioMérieux). Plates were left to dry for 15 min before application of Etests. Afterward, plates were transferred into anaerobic jars and incubated for 48 h at 37°C. Bio-Rad GelDoc XR (Bio-Rad Laboratories) was used to make images of the Etest results.

### RT-QPCR

Total RNA was isolated from 1 × 10^9^ cells in the mid-log phase (OD_600_ of 0.4–0.6) using the GeneJet RNA Purification Kit (Thermo Scientific) according to manufacturer instructions. Isolated RNA was diluted in nuclease-free water to a concentration of 10 ng μl^−1^ and stored at −20°C until further processing. RT-qPCR was performed in a CFX Connect Real-Time System (Bio-Rad) using Luna Universal One-Step RT-qPCR kit (New England Biolabs) in low profile, non-skirted 96-well plates sealed with optically clear adhesive seal sheets (Thermo Scientific). The reaction mixture (20 μl per reaction) contained 10 μl of Luna Universal One-Step reaction mix (2 ×), 1 μl of Luna WarmStart RT enzyme (20 ×), 0.8 μl of forward/reverse primer (10 μM), 50 ng of RNA (5 μl of 10 ng μl^−1^), and 2.4 μl of nuclease-free water. The RT-qPCR thermocycling protocol included one reverse transcription step at 55°C for 10 min, one initial denaturation cycle at 95°C for 1 min, 45 denaturation cycles at 95°C for 1 s, and 45 extensions cycles at 60°C for 30 s (+ plate read). At the end of the RT-qPCR run, melting curves were determined by stepwise (0.5°C per 5 s) increases in temperature from 60 to 95°C. All mRNA abundancies were measured in at least three biological experiments in triplicates. The *rpoD* (Wakimoto et al., 2013) and *gapdh* (Allen et al., 2019) genes were used as internal standards for normalization in order to calculate relative mRNA abundancies. Target mRNAs were *hmuY* (BF638R_1099), *feoAB* (BF638R_1421), and *nimA* (A7J11_00926). Primer efficiencies and mRNA abundancies were calculated along the lines of published methods (Vandesompele et al., 2002; Hellemans et al., 2007). All primers used are given in Supplementary Table 1.

### Statistical Analysis

Statistical analyses, including tests and the calculation of confidence intervals, were performed using GraphPad Prism 9 software. Details are given in the respective figure legends. The threshold of significance chosen was *p* < 0.05 or *q* < 0.05, the latter indicating the maximal false discovery rate.

## Results and Discussion

### PFOR Activity Is Strongly Upregulated in Strain 638R When Harboring a *nim* Gene

Due to the wide absence of a correlation of *nim* expression levels and the degree of metronidazole resistance as observed in a previous study (Leitsch et al., 2014), we hypothesized that Nim proteins might not act as nitroreductases which detoxify metronidazole but, rather, through an indirect mechanism. We further argued that this mechanism might include the modulation of the activities of metronidazole activating enzymes. Pyruvate:ferredoxin oxidoreductase (PFOR) is a central metabolic enzyme in most anaerobes (Narikawa, 1986) which catalyzes the decarboxylation of pyruvate to acetyl-CoA *via* the electron carrier protein ferredoxin. As reduced ferredoxin is a potent metronidazole-reducing factor *in vitro* (reviewed in Leitsch, 2019), the PFOR/ferredoxin couple has been repeatedly proposed as the major activation pathway of metronidazole and other nitroimidazoles, also in *B*. *fragilis* (Narikawa et al., 1991). This notion received additional support by the observed downregulation of PFOR and ferredoxin in metronidazole-resistant microbes (reviewed in Leitsch, 2019). We wanted to test whether the presence of *nim* genes has an effect on PFOR activity in *B*. *fragilis* and determined PFOR activities in strain 638R and two daughter strains which had received an episomal *nim* gene through transconjugal transfer of natural plasmids from clinical strains: 638R *nimA* (harboring pI417) and 638R *nimE* (harboring pBF388c). Both strains display reduced susceptibility to metronidazole, with an MIC of 1.5 μg ml^−1^ metronidazole in case of 638R *nimA* (Paunkov et al., 2022) and 8 μg ml^−1^ in case of 638R *nimE* (Sóki et al., 2006), respectively. In pI417, the *nimA* gene is transcribed from a promoter in the IS1168 element and is preceded by a transposase gene. Otherwise, the pl417 carries genes for plasmid maintenance (*repA, mobA*, and *mobB*), two genes of unknown function and an *ermF* resistance gene which, however, is obviously not transcribed or inactive because it does not confer resistance to erythromycin (Paunkov et al., 2022). With the exception of the *nimA* gene, no other factor encoded in pI417 can be reasonably linked to metronidazole resistance. In pBF388c, the *nimE* gene is transcribed from a promoter in the IS*Bf* 6 element and is also preceded by a transposase gene. Otherwise, pBF388c has not been characterized so far.

**Figure 1:**
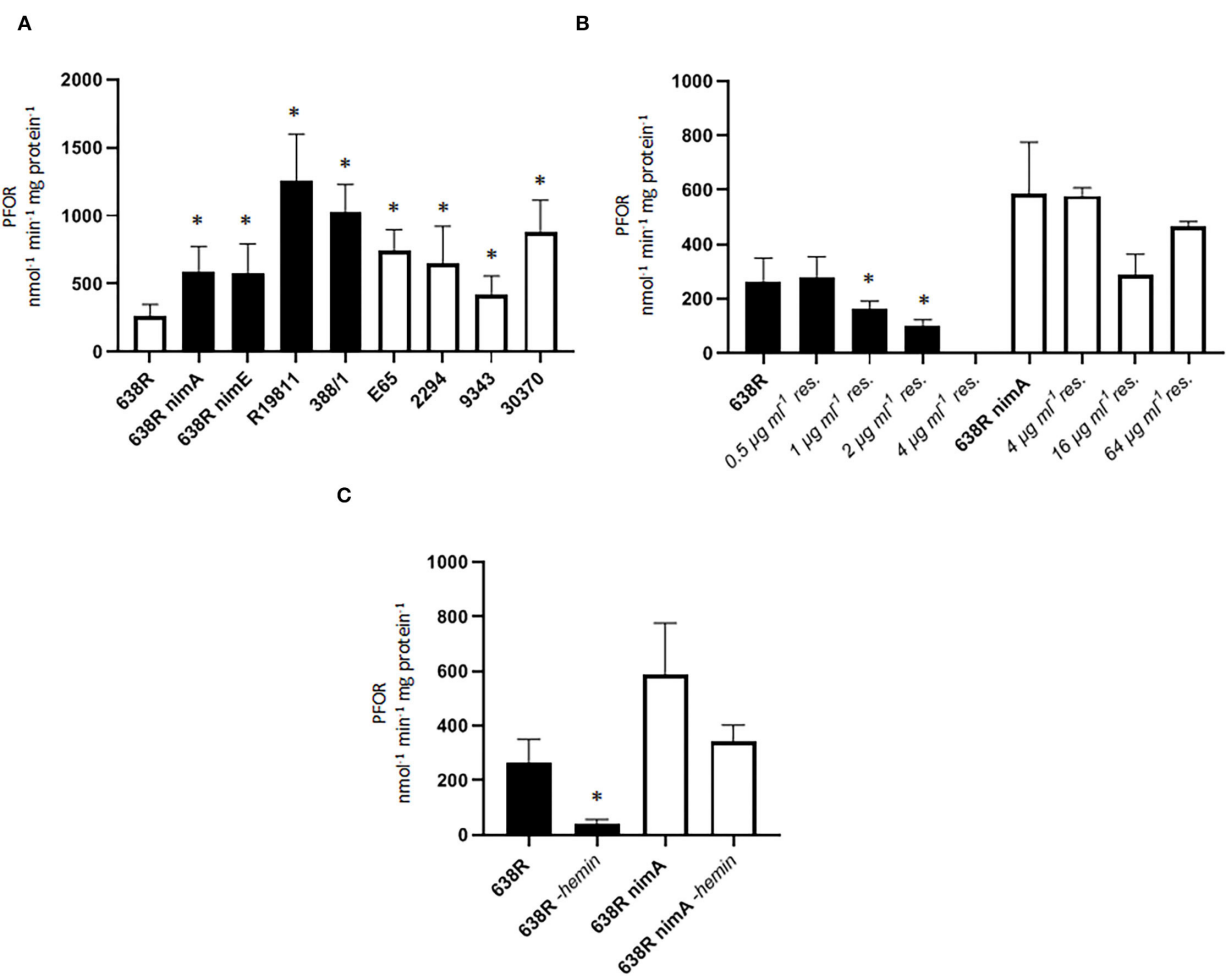
PFOR activity in *B*. *fragilis*. **(A)** PFOR activity in several *B*. *fragilis* strains. Black bars indicate strains with reduced metronidazole susceptibility (638R *nimA* and *nimE*) and metronidazole-resistant clinical isolates (R19811, 388/1). The MICs for metronidazole: 638R, 0.25 μg ml^−1^ (Paunkov et al., 2022); 638R *nimA*, 1.5 μg μg ml^−1^ (Paunkov et al., 2022); 638R *nimE*, 8 μg ml^−1^ (Sóki et al., 2006); R19811, 48 μg ml^−1^ (Paunkov et al., 2022); 388/1, 256 μg ml^−1^ (Paunkov et al., 2022); E65, 0.25 μg ml-1 (this study), 2294, 0.50 μg ml^−1^ (this study); 30370, 0.38 μg ml^−1^ (this study); NCTC9343, 0.50 μg ml^−1^ (this study). Number of measurements: 16 (638R), 15 (638R *nimA*), 11 (638R *nimE*), 10 (R19811), 3 (388/1), 6 (E65), 8 (2294), 6 (NCTC 9343), 6 (30370). Non-parametric test (Kruskal–Wallis test on ranks) with multiple comparisons (Two-stage step-up method by Benjamini, Krieger and Yekutieli): * >638R (*q* < 0.05). **(B)** Induction of metronidazole resistance leads to shutoff of PFOR activity in resistant 638R (black bars) but not in resistant 638R *nimA* (white bars). Number of measurements: non-induced strains see A., all induced strains were measured three times. Non-parametric test (Kruskal–Wallis test on ranks) with multiple comparisons (Two-stage step-up method by Benjamini, Krieger and Yekutieli): *smaller than 638R (*q* < 0.05). **(C)** Strong decrease in PFOR activity in 638R but not 638R *nimA* upon withdrawal of hemin from the growth medium. Number of measurements: 638R and 638R *nimA* see **(A)**, all others were measured three times. Unpaired *t*-test (two-tailed): *638R-hemin <638R (*p* < 0.05).

We hypothesized that PFOR activity would be downregulated in the strains with a *nim* gene because tolerance to metronidazole has been repeatedly reported to be inversely proportional to PFOR activity (reviewed in Leitsch, 2019). In contrast to our expectation, however, PFOR activity proved to be strongly increased in 638R *nimA* and 638R *nimE* as compared to the parental strain 638R (Figure 1A). PFOR activity was even more pronounced in the two highly metronidazole-resistant clinical isolates R19811 and 388/1, the former not harboring any *nim* gene (Terhes et al., 2001) and the latter carrying the *nimE* gene on plasmid pBF388c (Sóki et al., 2006). R19811 displayed the highest activity of all strains tested amounting to an ≈7-fold rate as compared to 638R. In four more *B*. *fragilis* strains (E65, 2294, NCTC9343, and 30370), all of which are fully susceptible to metronidazole (data not shown), PFOR activities ranged between the two extremes. These results show that, first, *nim* genes do indeed have an impact on cellular physiology and, second, that there is no causative correlation between PFOR activity and metronidazole resistance.

We next wanted to survey PFOR activity during the development of increasing resistance and induced higher levels of metronidazole resistance in 638R and 638R *nimA* by passaging cells on plates with metronidazole concentrations doubling with every passage (ranging from initial 0.5 μg ml^−1^ to a final 64 μg ml^−1^). Interestingly, strain 638R had completely lost PFOR activity already at 4 μg ml^−1^ (Figure 1B) whereas PFOR activity in 638R *nimA* was practically unaffected even at the highest concentration tested. This suggests that *nimA* enables a fundamentally different course to metronidazole resistance, at least if resistance is induced *in vitro*. Importantly, strain R19811 (Figure 1A) shows the highest PFOR activity of all strains tested although it lacks a *nim* gene, which underlines that development of resistance *in vivo* is different from that *in vitro*.

PFOR heavily relies on iron levels because it harnesses iron–sulfur clusters for activity (Menon and Ragsdale, 1997). Accordingly, PFOR is inactive or downregulated upon iron depletion in some anaerobes, for example, in the trichomonad parasites *Tritrichomonas foetus* and *Trichomonas vaginalis* (Vanáčová et al., 2001; Leitsch et al., 2009). On the contrary, induction of metronidazole resistance also leads to the loss of PFOR activity in trichomonads (reviewed in Leitsch, 2019), suggesting a correlation between *in vitro* metronidazole resistance, PFOR activity, and intracellular iron levels. In order to test whether this is also the case in *B*. *fragilis* we omitted hemin, which constitutes the primary iron source in growth media for *B*. *fragilis*, from agar plates and liquid broth and measured PFOR activity in 638R and 638R *nimA* cells. PFOR activity in 638R was again steeply reduced, whereas PFOR in 638R *nimA* was far less affected (Figure 1C). This result suggested that the *nimA*-harboring strain is not affected by the iron depletion comparatively to the parental strain.

**Figure 2:**
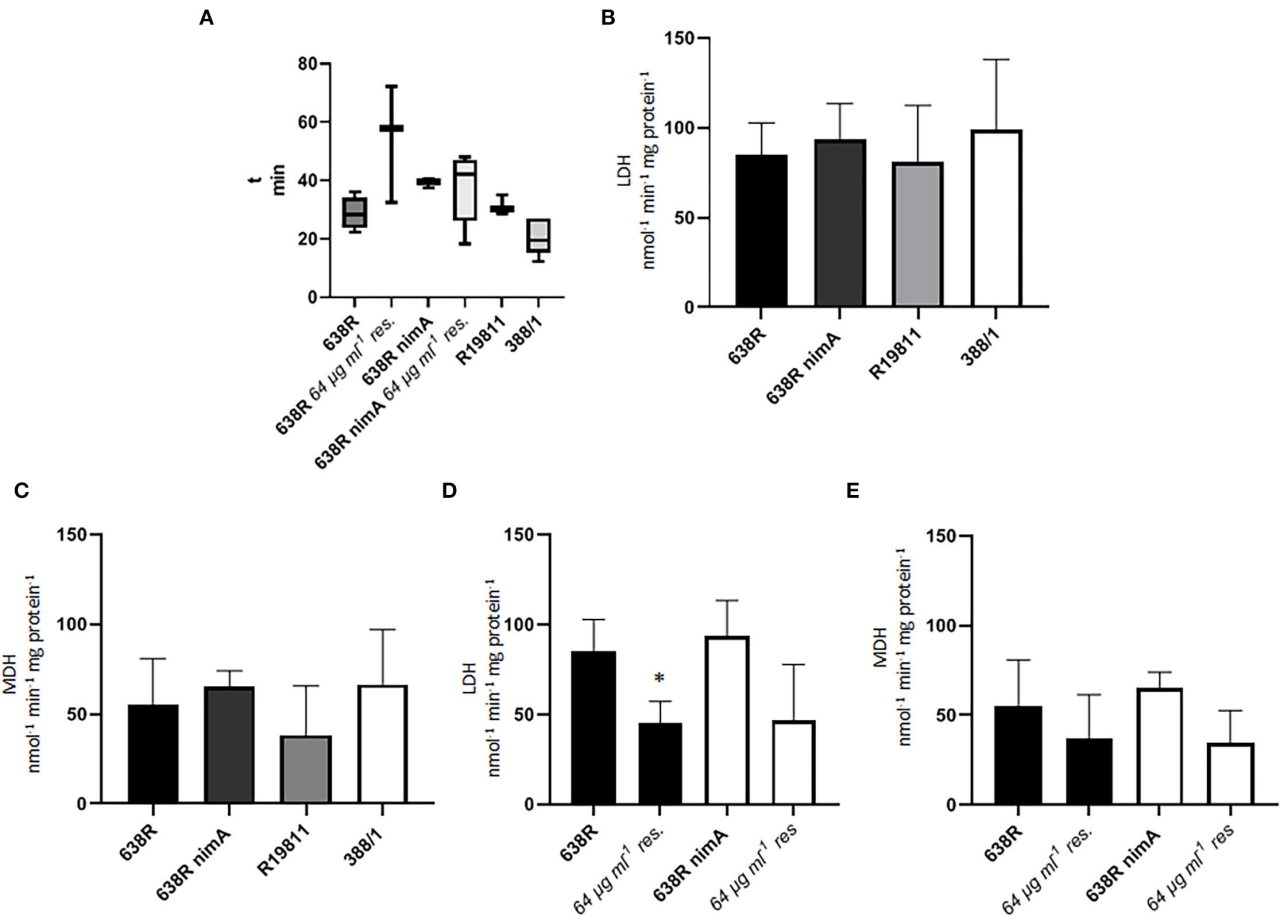
Activities of important metabolic enzymes in *B*. *fragilis*. **(A)** Fumarate reductase (FR) activity in 638R, metronidazole-resistant 638R (resistant to 64 μg ml^−1^ of metronidazole), 638R *nimA*, metronidazole-resistant 638R *nimA* (64 μg ml^−1^ of metronidazole), R19811 (resistant to 48 μg ml^−1^ of metronidazole), and 388/1 (resistant to 256 μg ml^−1^ of metronidazole). The time necessary to bleach benzyl viologen in the reaction mixture is inversely proportional to FR activity. All strains were measured at least three times. **(B)** Lactate dehydrogenase (LDH) activity in 638R, 638R *nimA*, R19811, and 388/1. All strains were measured three times. **(C)** Malate dehydrogenase (MDH) activity in 638R, 638R *nimA*, R19811, and 388/1. All strains were measured three times. **(D)** LDH activity in susceptible and resistant 638R and 638R *nimA*, respectively. All strains were measured three times. Unpaired *t*-test (two-tailed): *638R 64 μg ml^−1^ <638R (*p* < 0.05). **(E)** MDH activity in susceptible and resistant 638R and 638R *nimA*, respectively. All strains were measured three times.

Another central metabolic enzyme in *B*. *fragilis* is fumarate reductase (FR) which depends on heme as a cofactor and which reduces fumarate to succinate (Baughn and Malamy, 2003). FR activity was only found to be slowed down in resistant 638R (Figure 2A), suggesting that the *nimA* gene also has a stabilizing effect on FR activity. Next, we assayed the activities of lactate dehydrogenase (LDH) and malate dehydrogenase (MDH), two other central metabolic enzymes in *B*. *fragilis* and many other anaerobes. Especially LDH has been repeatedly suggested to be involved in metronidazole resistance because it can metabolize excess pyruvate to lactate in cells with decreased PFOR activity. Accordingly, LDH was found upregulated in metronidazole-resistant *T*. *vaginalis* (reviewed in Kulda, 1999) and *B*. *fragili*s (Narikawa et al., 1991). In contrast to these previous findings, however, we could not detect any obvious impact of metronidazole resistance on LDH and MDH activities in the strains 638R, 638R *nimA*, R19811, and 388/1 (Figures 2B,D). Furthermore, also in 638R and 638R *nimA* cells with induced metronidazole resistance, no increase in LDH and MDH activities could be observed (Figures 2C,E). Rather, LDH and MDH were slightly downregulated in the resistant cells, although this was statistically significant only in case of LDH in resistant 638R.

### *nimA* Ensures a Far Higher Survivability in the Presence of Oxygen After the Induction of Metronidazole Resistance

**Figure 3:**
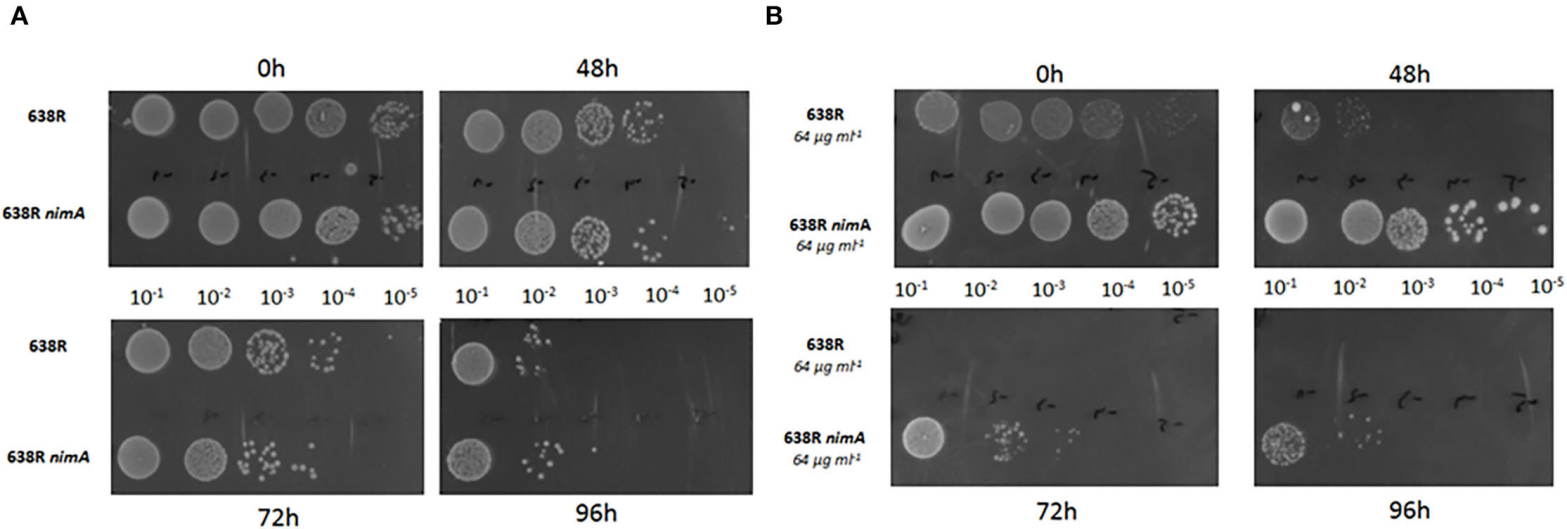
Oxygen tolerance of *B*. *fragilis* strains. **(A)** Liquid cultures of 638R, 638R *nimA*, and 638R *nimE* at an OD_600_ of 0.5 were diluted (10^−1^, 10^−2^, 10^−3^, 10^−4^, and 10^−5^) and spotted on BHI agar supplemented with hemin. Cells were incubated in the presence of air in an incubator at 37°C for the time intervals as indicated. **(B)** Identical procedure as in **(A)**, but with metronidazole-resistant cells (64 μg ml^−1^). All experiments were performed three times. The images of all oxygen tolerance assays are shown in Supplementary Figure 1.

Metronidazole has been repeatedly shown to impair the antioxidant defense (reviewed in Leitsch, 2019), so it was hypothesized that *nimA* might confer higher tolerance to metronidazole indirectly by conferring a higher tolerance to oxidative stress. When we exposed 638R and 638R *nimA* to oxygen for extended periods of time (48, 72, and 96 h), the daughter strain did not perform differently from the parental strain (Figure 3A). After induction of metronidazole resistance, however, 638R was much more susceptible to oxygen than 638R *nimA* with hardly any cells surviving after 48 h of exposure to air (Figure 3B). In contrast, 638R *nimA* cells were viable for at least 72 h under the same conditions even up to 96 h (Figure 3B). It is also important to note that resistant 638R also grew much slower than the daughter strains with the *nimA* gene, as deducible from the much smaller colony sizes (Figure 3B).

### Resistant 638R Has a Much Lower Thioredoxin Reductase (TrxR) Activity Than the Resistant Daughter Strain 638R *nimA*

**Figure 4:**
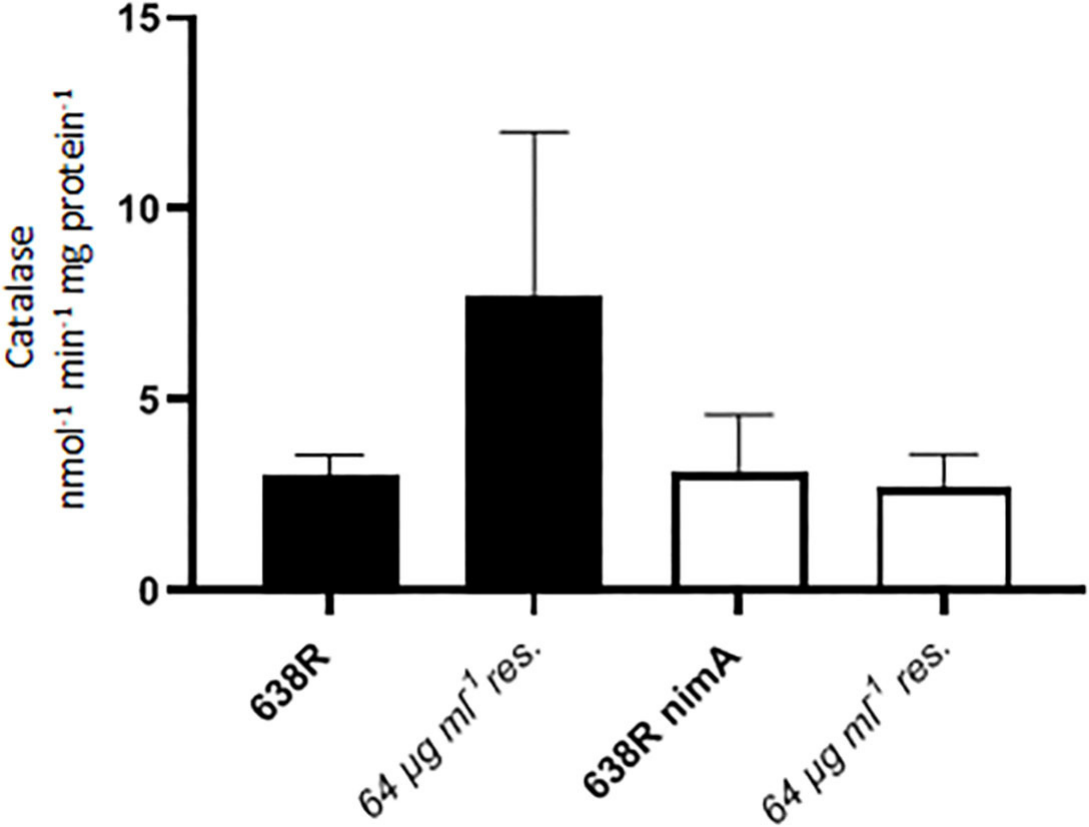
Catalase activity in 638R and 638R *nimA* before and after induction of high-level metronidazole resistance (64 μg ml^−1^). All strains were measured three times.

Subsequently, it was tested whether the enhanced sensitivity of oxygen in resistant 638R was caused by downregulation of antioxidant enzymes. In a previous study, catalase had already been found fully active in the resistant clinical isolates R19811 and 388/1 (Paunkov et al., 2022). Accordingly, catalase activity was also widely unchanged in metronidazole-resistant 638R and 638R *nimA* as compared to the respective pre-resistant cells (Figure 4). In fact, catalase was even higher in resistant 638R in some measurements, but not reproducibly so. As catalase depends on heme as a cofactor for activity, this result shows that *in vitro* metronidazole resistance does not automatically result in a reduction in iron-dependent enzyme activities as previously observed with PFOR. Also, superoxide dismutase (SOD) activity was unchanged after induction of high-level resistance in 638R and 638R *nimA* (Table 1). It is interesting to note, however, that SOD activity was clearly higher in the resistant clinical strains R19811 and 388/1 (Table 1).

**Table 1:** Superoxide dismutase activity in cell extracts of *B*. *fragilis* strains.

**Strains**	**SOD activity (units)**
638R	246
638R 64 μg ml^−1^	270
638R *nimA*	222
638R *nimA* 64 μg ml^−1^	208
R19811	390
388/1	480

*All strains were measured three times*.

**Figure 5:**
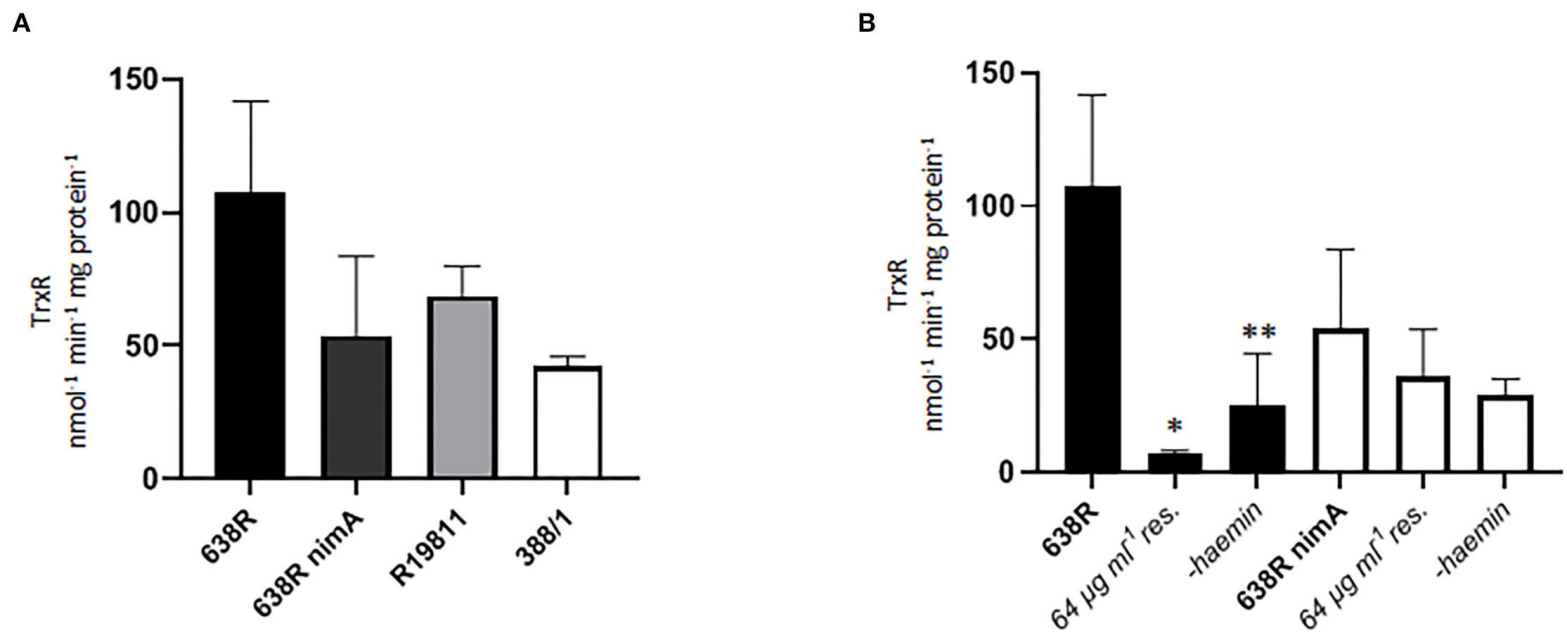
Thioredoxin reductase (TrxR) activity in *B*. *fragilis*. **(A)** TrxR activity in cell extracts of 638R, 638R *nimA*, R19811, and 388/1. All strains were measured three times. **(B)** TrxR activity in 638R and 638R *nimA* before and after induction of metronidazole resistance (64 μg ml^−1^), and after withdrawal of hemin from the growth medium. All strains were measured three times. Unpaired *t*-test (two-tailed): *638R 64 μg ml^−1^ <638R (*p* < 0.05), **638R-hemin <638R (*p* < 0.05).

Finally, we assayed the activity of thioredoxin reductase (TrxR), a central redox enzyme in most organisms including *B*. *fragilis* (Rocha et al., 2007; Paunkov et al., 2021), in the strains 638R, 638R *nimA*, R19811, and 388/1. TrxR is a central redox enzyme which regulates the activity of several key antioxidant enzymes such as peroxiredoxin or methionine sulfate reductase *via* thioredoxin, its principal substrate. Accordingly, TrxR activity is strongly upregulated upon exposure to oxygen in *B*. *fragilis* (Paunkov et al., 2021). Activities were rather similar in all strains tested, with 638R displaying the highest activity, but not consistently so in all measurements (Figure 5A). However, in 638R with induced resistance, TrxR activity was dramatically decreased to <10% of the original level whereas TrxR in 638R *nimA* was not affected to a significant degree (Figure 5B). A sharp decrease in TrxR activity had been observed before in *T*. *vaginalis* with high-level metronidazole resistance induced *in vitro* (Leitsch et al., 2009). This had been caused by a lack of FAD cofactor in the resistant cell line because TrxR activity could be widely restored upon addition of FAD to cell extracts when performing the measurements (Leitsch et al., 2009). The addition of FAD (5 μM) to extracts of resistant 638R, however, did not increase TrxR activity (data not shown), suggesting that the decrease in TrxR activity is caused by a different mechanism. We argued that, accordingly to PFOR activity (Figure 1B), TrxR activity might also be linked to iron levels and repeated the measurements with cells grown without hemin. A similar reduction in TrxR activity as seen before with PFOR (Figure 1C) was observed in 638R but not in 638R *nimA* under these conditions (Figure 5B).

### Iron Import Is Strongly Downregulated in Resistant 638R but Not in Resistant 638R *nimA*

**Figure 6:**
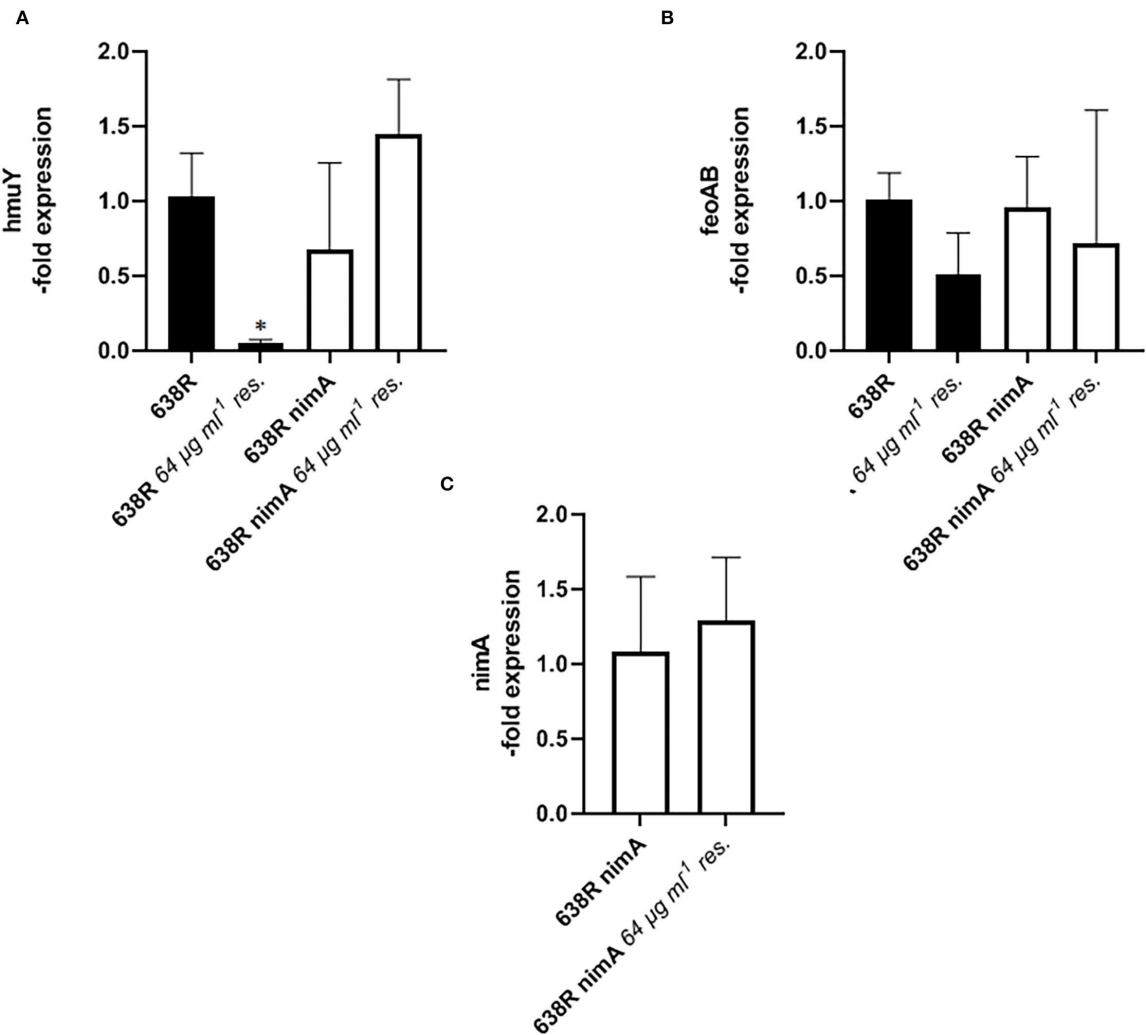
RT-qPCR analysis of gene expression in *B*. *fragilis*. **(A)** mRNA levels of hemin import protein HmuY in 638R and 638R *nimA* before and after induction of metronidazole resistance (64 μg ml^−1^). All strains were measured three times in triplicate. Unpaired *t*-test (two-tailed): *638R <638R 64 μg ml^−1^ (*p* < 0.05). **(B)** mRNA levels of iron import factor FeoAB in 638R and 638R *nimA* before and after induction of metronidazole resistance (64 μg ml^−1^). All strains were measured three times in triplicate. **(C)** Expression levels of *nimA* 638R *nimA* before and after induction of high-level metronidazole resistance (64 μg ml^−1^). All strains were measured three times in triplicate.

**Figure 7:**
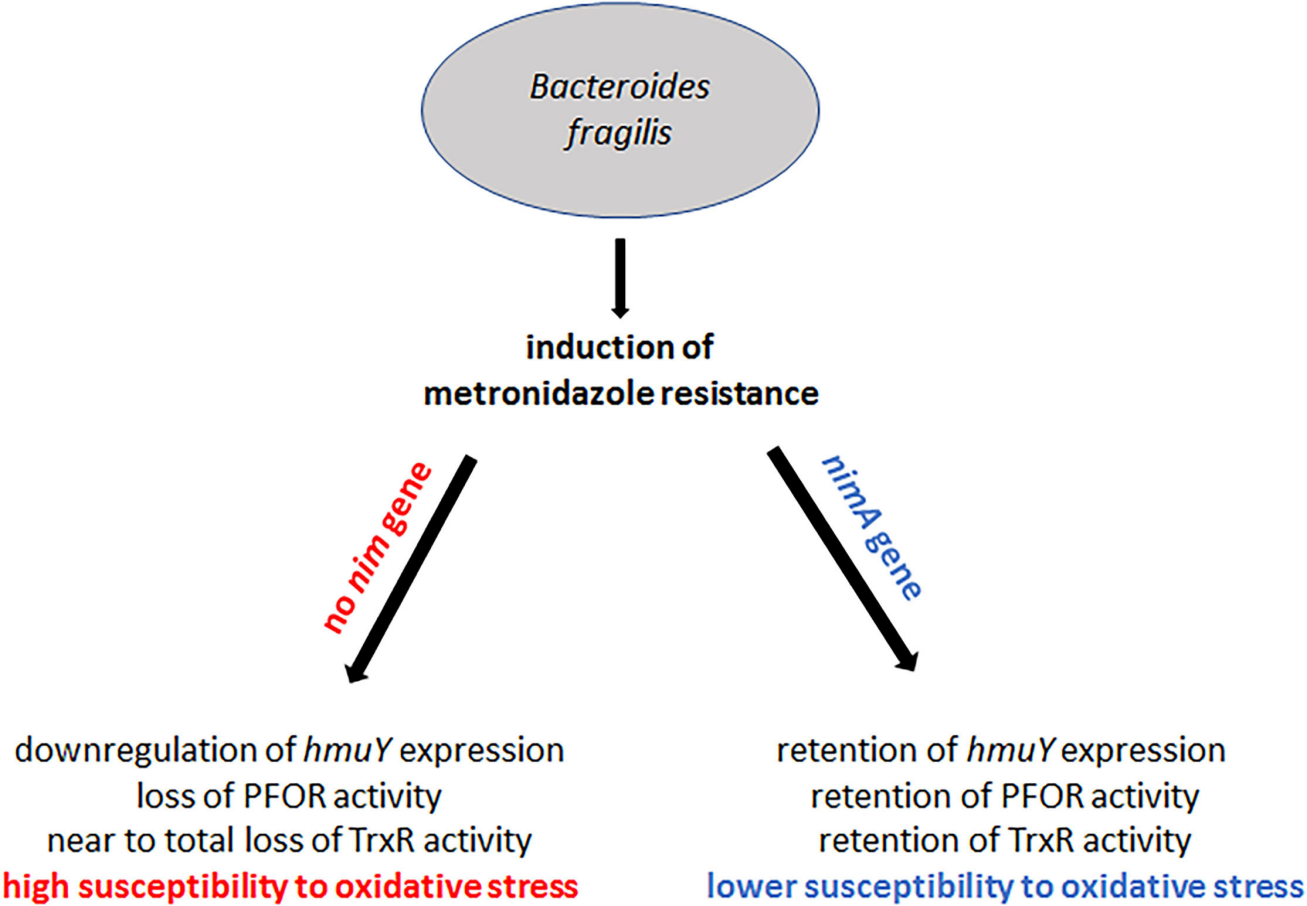
Summary of the results on the effect of *nimA* on the development of metronidazole resistance in *B*. *fragilis*. High-level metronidazole resistance can be induced in *B*. *fragilis* with and without a *nimA* gene. In the absence of a *nimA* gene, iron import is downregulated through downregulation of *hmuY* expression whereas in the presence of *nimA* expression of *hmuY* is retained at the original level. As a consequence, PFOR and TrxR are downregulated or even shut off in *B*. *fragilis* without *nimA*. Downregulation of TrxR might further lead to the high susceptibility to oxygen of highly resistant 638R without a *nimA* gene. In highly resistant 638R *nimA*, however, the levels of PFOR and TrxR are affected to a far lesser extent, consequently leading to a higher oxygen tolerance as compared to highly resistant 638R.

The parallelism of decreased PFOR and TrxR activities in metronidazole-resistant 638R and hemin-deprived 638R suggested that metronidazole resistance might be linked to impaired iron import. In contrast, the presence of the *nimA* gene seems to ensure the preservation of normal intracellular iron levels, even after the induction of high-level resistance and omission of hemin from the growth medium. We therefore hypothesized that either the ferrous iron transporter FeoAB (Veeranagouda et al., 2014) or the hemin-uptake protein HmuY (Olczak et al., 2008) might be downregulated in resistant 638R but not in resistant 638R *nimA* and performed a RT-qPCR analysis of the respective genes in both strains. Indeed, the level of *hmuY* mRNA was decreased in resistant 638R cells to about 5% of the level found in susceptible 638R cells (Figure 6A). In contrast, *hmuY* mRNA was not downregulated in resistant 638R *nimA*. The *nimA* gene had, however, no effect on *hmuY* mRNA levels prior to the induction of high-level resistance (Figure 6A). The level of *feoAB* mRNA was very similar in all strains tested, if somewhat lower in resistant 638R than in the susceptible parent strain (Figure 6B). We also determined expression levels of *nimA* in the 638R *nimA* prior to and after induction of high-level resistance. As observed earlier at the protein level (Leitsch et al., 2014), the mRNA level of the *nimA* gene was not or only slightly increased in resistant cells (Figure 6C), again suggesting a different role of Nim proteins than that of nitroreductases. Rather, NimA seems to positively affect iron uptake in *B*. *fragilis via* the hemin import protein HmuY (Figure 7).

## Conclusion

Metronidazole resistance is a complex phenomenon in most anaerobes, with *Helicobacter pylori* and its close relative *Campylobacter jejuni* being rare exceptions in which resistance is caused rather swiftly through the loss of nitroreductase RdxA (Jenks et al., 1999; Ribardo et al., 2010). Usually, metronidazole resistance is accompanied by wide-ranging physiological changes, especially if resistance is induced in the laboratory (reviewed in Leitsch, 2019). This is also true for *B*. *fragilis* in which metronidazole resistance had been found to be correlated with a loss of PFOR, an enzyme which has been proposed as the major metronidazole-reducing factor in anaerobes (Narikawa, 1986; Narikawa et al., 1991). It was later questioned, however, if this correlation is indeed causative of resistance (Diniz et al., 2004). Here, we clearly show that impairment of PFOR activity is caused likewise by the induction of metronidazole resistance and by decreasing iron levels in the growth medium, but only in *B*. *fragilis* 638R without a *nim* gene. In 638R *nimA*, PFOR remained active even after having attained high-level metronidazole resistance and also after withdrawal of hemin from growth media. As PFOR activity was found to be even the highest in the metronidazole-resistant isolates R19811 and 388/1, the former without a *nim* gene, among several strains tested, we conclude that PFOR activity and metronidazole resistance are not causally linked. Probably, downregulation of PFOR activity, but also of fumarate reductase, is a consequence of decreased import of hemin/iron due to lower expression levels of the hemin import protein HmuY (Olczak et al., 2008) in metronidazole-resistant 638R. The mRNA levels of ferrous iron transporter FeoAB were unchanged in resistant 638R which was surprising because deletion of the *feoAB* gene had been found to result in low-level resistance (Veeranagouda et al., 2014). As FeoAB is involved in the release of iron from heme (Rocha et al., 2019), however, both proteins act within the same pathway and the deficiency of either has a similar outcome. Importantly, downregulation of *hmuY* expression was not observed in resistant 638R *nimA*, suggesting that the presence of a *nim* gene leads to an alternative development of metronidazole resistance. Accordingly, resistant 638R *nimA* was much more tolerant to oxygen than resistant 638R. Catalase and SOD were similarly active in 638R and 638R *nimA*, both metronidazole-susceptible and resistant, but TrxR activity was greatly decreased in resistant 638R. As observed earlier with PFOR, a similar effect on enzyme activity was observed after withdrawing hemin from the growth medium, again only in the absence of the *nimA* gene. Since TrxR is a central redox enzyme, we propose that the TrxR deficiency in resistant 638R cells contributes to the high oxygen sensitivity observed.

Taken together, the results of this study provide further evidence that Nim proteins are unlikely to act only or primarily as nitroreductases. This notion is further corroborated by the unchanged *nimA* mRNA level in resistant 638R *nimA* as compared to the parent strain which only displays reduced susceptibility (638R *nimA*). In an earlier study, it had already been shown that Nim protein levels are not elevated in high-level resistant strains (Leitsch et al., 2014). Rather, Nim proteins seem to act through a more indirect mechanism which prevents the impairment of iron import as seen with 638R cells without a *nim* gene, leading to a much better physiological performance overall. This might explain why high-level resistance can be much easier and faster induced in strains with a *nim* gene than without (Löfmark et al., 2005; Leitsch et al., 2014). The underlying mechanism, however, remains to be discovered, and pertinent studies are currently being undertaken in our laboratory.

## Data Availability Statement

The original contributions presented in the study are included in the article/Supplementary Material, further inquiries can be directed to the corresponding author/s.

## Author Contributions

AP performed the experiments, conceived the experiments, and analyzed the data. JS conceived the experiments and analyzed the data and DL conceived the experiments, analyzed the data, and wrote the manuscript. All authors contributed to the article and approved the submitted version.

## Funding

This research was funded in whole, or in part, by the Austrian Science Fund (FWF) [grant number I 4234]. For the purpose of open access, the author has applied a CC BY public copyright license to any Author Accepted Manuscript version arising from this submission. Further, JS was funded by grant ANN_130760 from the National Research, Development and Innovation Office of Hungary (NKFIH).

## Conflict of Interest

The authors declare that the research was conducted in the absence of any commercial or financial relationships that could be construed as a potential conflict of interest.

## Publisher's Note

All claims expressed in this article are solely those of the authors and do not necessarily represent those of their affiliated organizations, or those of the publisher, the editors and the reviewers. Any product that may be evaluated in this article, or claim that may be made by its manufacturer, is not guaranteed or endorsed by the publisher.
