# Supplementary material for: Modulation of Iron Import and Metronidazole Resistance in Bacteroides fragilis Harboring a nimA Gene
Source: Front Microbiol. 2022 Jun 9;13:898453. doi: 10.3389/fmicb.2022.898453 (PMC9218692; doi:10.3389/fmicb.2022.898453)
Supplement: Supplementary file 2 [file Data_Sheet_1.PDF]

Supplementary Figure 1
Survival assay

Three biological replicates with the following strains

- 638R
- 638R nimA
- 638R nimE

Experimental setup is described in the main text

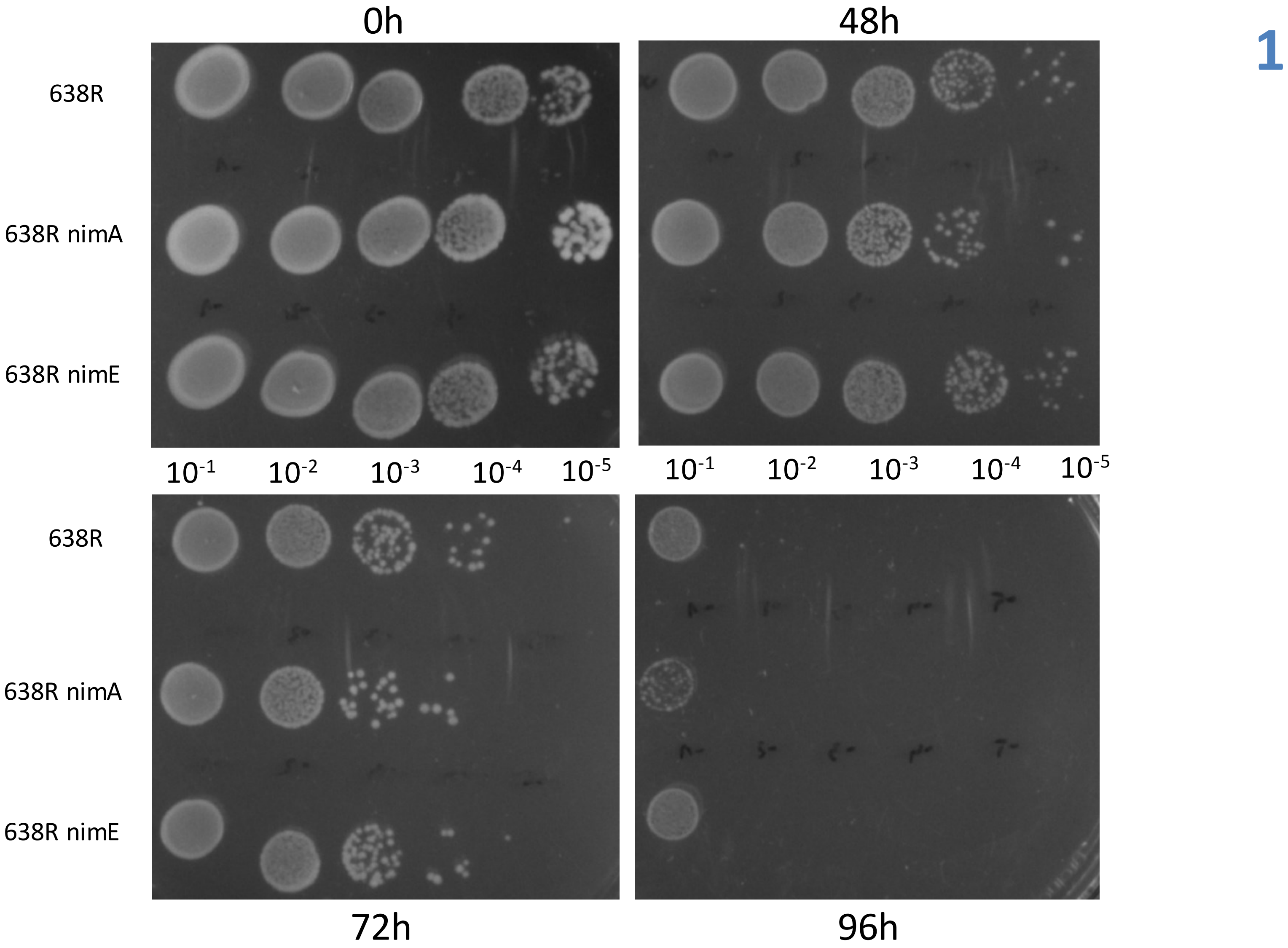
0h
48h
1
638R
638R nimA
638R nimE
10-1
10-2
10-3
10-4
10-5
10-1
10-2
10-3
10-4
10-5
638R
638R nimA
638R nimE
72h
96h

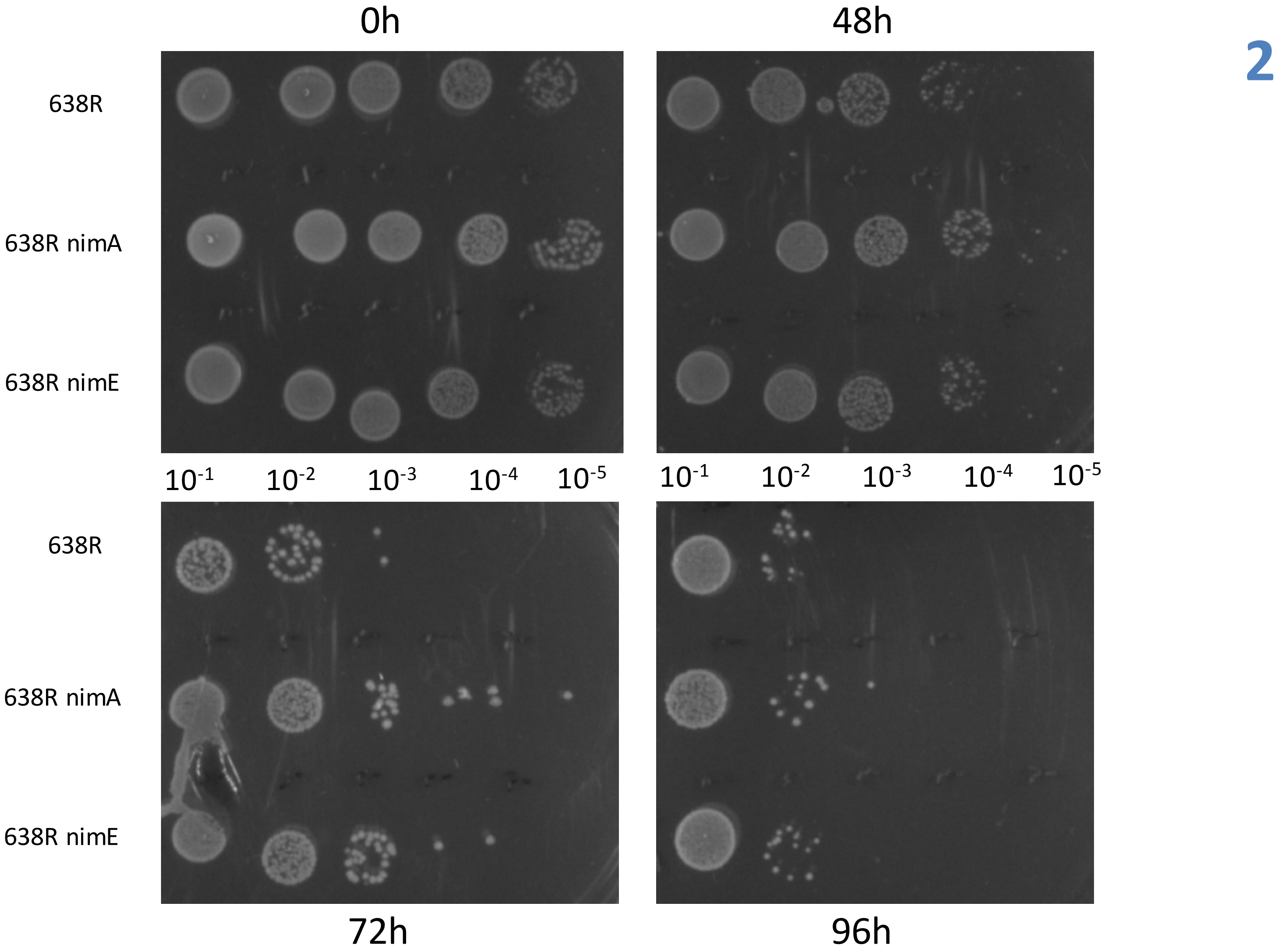
2
0h
48h
638R
638R nimA
638R nimE
10-1 10-2 10-3 10-4 10-5
10-1 10-2 10-3 10-4 10-5
638R
638R nimA
638R nimE
72h
96h

3

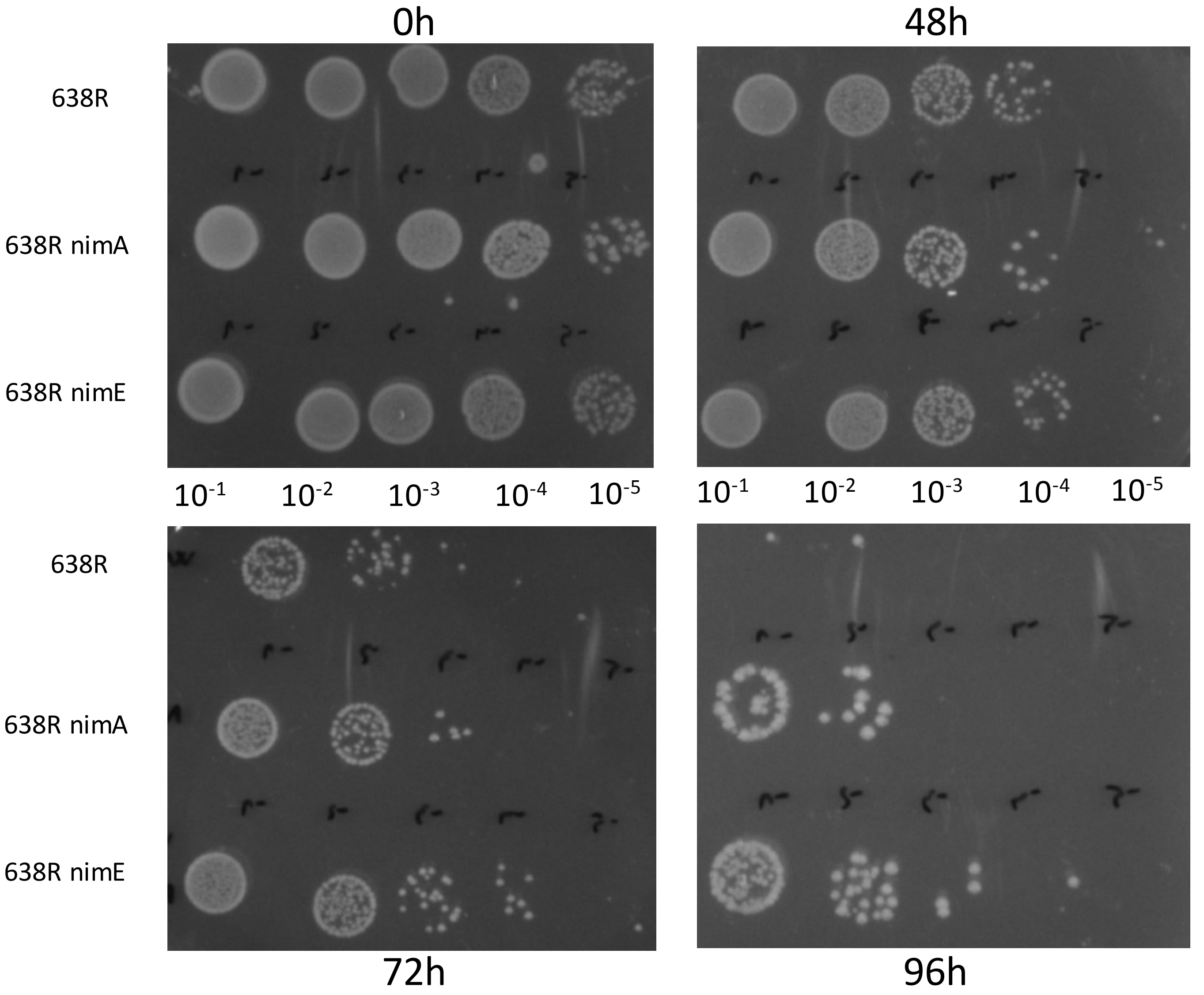
0h
48h
638R
638R nimA
638R nimE
10-1 10-2 10-3 10-4 10-5
10-1 10-2 10-3 10-4 10-5
638R
638R nimA
638R nimE
72h
96h

Three biological replicates with the following strains

- 638R 64 µg ml^{-1}
- 638R nimA 64 µg ml^{-1}
- Bf 388/1

Experimental setup is described in the main text

1

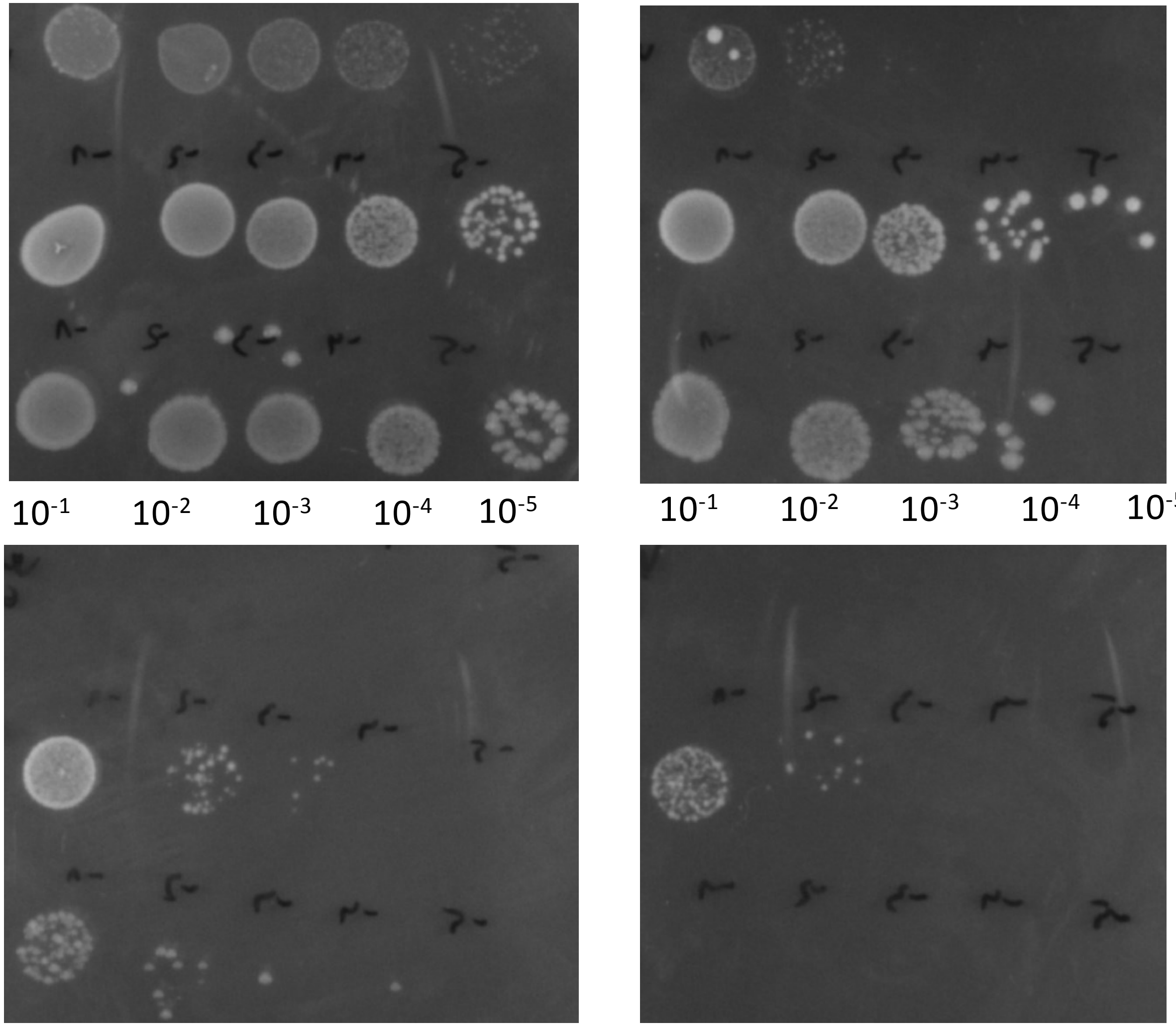

0h
48h
638R 64
638R NimA 64
388/1 NimE
10-1 10-2 10-3 10-4 10-5
10-1 10-2 10-3 10-4 10-5
638R 64
638R NimA 64
388/1 NimE
72h
96h

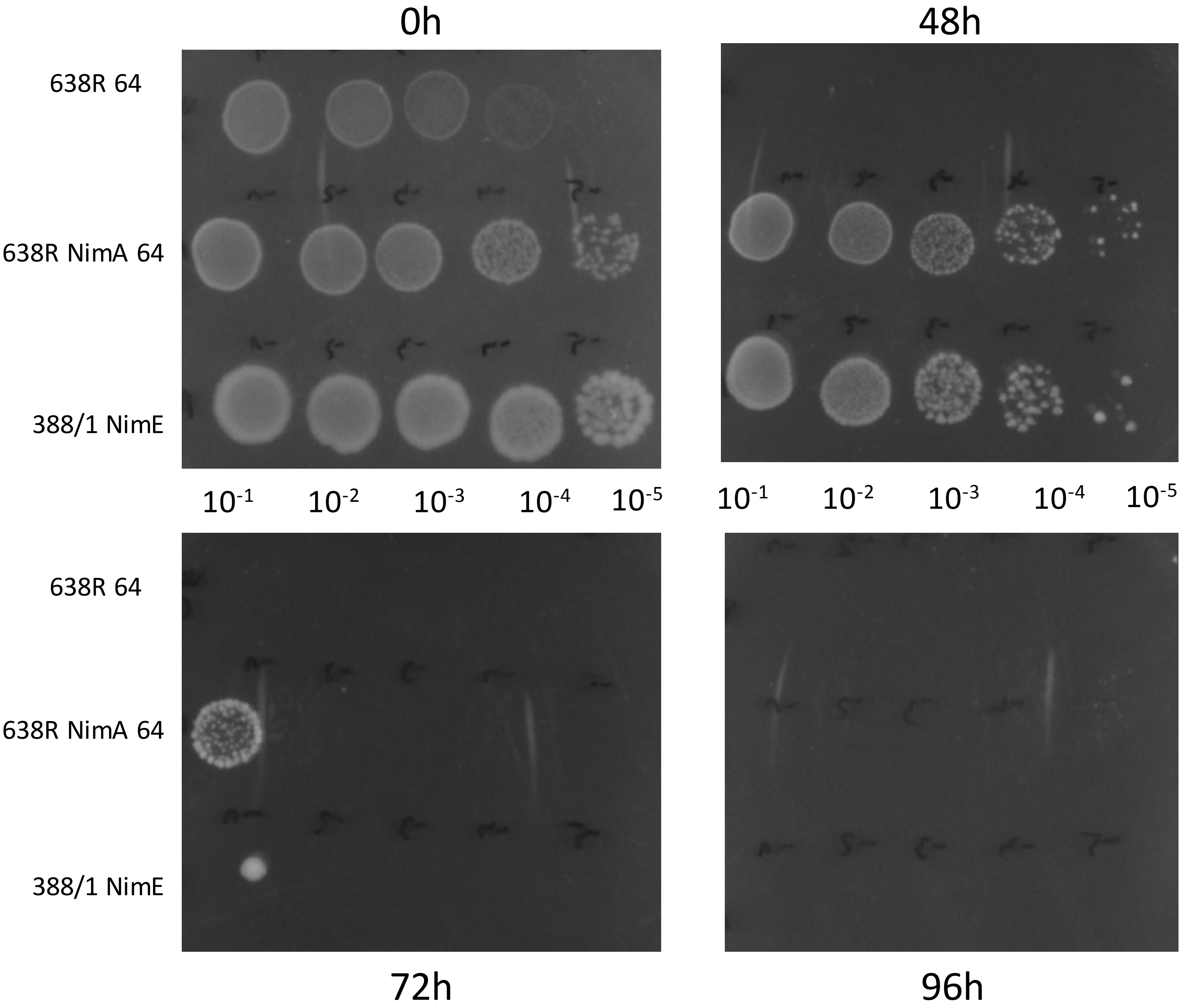
0h
48h
638R 64
638R NimA 64
388/1 NimE
10-1 10-2 10-3 10-4 10-5
10-1 10-2 10-3 10-4 10-5
638R 64
638R NimA 64
388/1 NimE
72h
96h

2

3

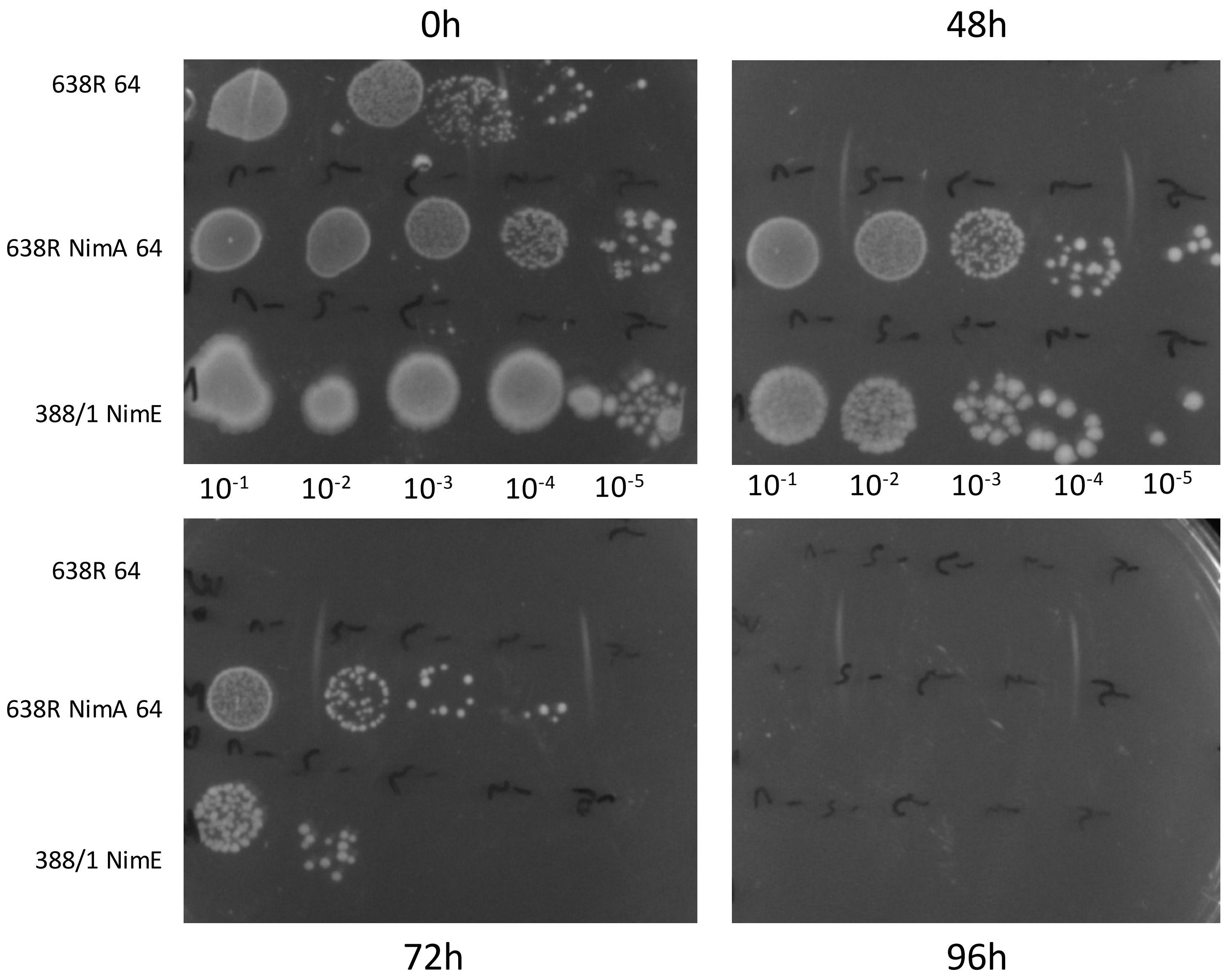
0h
48h
638R 64
638R NimA 64
388/1 NimE
10^{-1} 10^{-2} 10^{-3} 10^{-4} 10^{-5}
10^{-1} 10^{-2} 10^{-3} 10^{-4} 10^{-5}
638R 64
638R NimA 64
388/1 NimE
72h
96h
