# Supplementary material for: Modulation of Iron Import and Metronidazole Resistance in Bacteroides fragilis Harboring a nimA Gene
Source: Front Microbiol. 2022 Jun 9;13:898453. doi: 10.3389/fmicb.2022.898453 (PMC9218692; doi:10.3389/fmicb.2022.898453)
Supplement: Supplementary file 3 [file Data_Sheet_2.PDF]

Gene	Direction	Sequence
rpoD	Forward	5’ - TGG CAT TAG AGA AAC TGA CAC – 3’
	Reverse	5’ - CGA ATC CAC CAT ACA GCA TAA C – 3’
gapdh	Forward	5’ - GAA AAC ATC ATC CCG TCT TC – 3’
	Reverse	5’ - AGC CAT TGT AGC AGC TTT TT – 3’
nimA	Forward	5’ - GTA AAG CCC GTA TCC TGA CC – 3’
	Reverse	5’ - CGT CCC ACC TGT TAT TTC CC – 3’
nimE	Forward	5’ - TCC TTA TGC CGT TCC TCT C - 3’
	Reverse	5’ - CAC CAG CAA AGT CAT AGC C – 3’
hmuY	Forward	5’ - TTT ATC ATC ACC GCT TGC TC – 3’
	Reverse	5’ - CTC CAC CTT TTC CTT TGC C – 3’
feoAB	Forward	5’ - GAG ACT TTT ACC GAT AAC CAC C – 3’
	Reverse	5’ - GAT AAC TCC TCC TAC TCC ACC – 3’

Supplementary table 1. RT-qPCR primer sequences
